# Determinants of Pneumothorax and Alveolar Hemorrhage After CT-Guided Lung Biopsy

**DOI:** 10.3390/diagnostics16121848

**Published:** 2026-06-15

**Authors:** Amalia Constantinescu, Alessia-Stephania Roșian, Radu-Nicolae Căprariu, Ionel-Alin Muntean, Versavia Maria Ancușa, Alin Ciprian Nicola, Cristian Oancea, Diana Manolescu

**Affiliations:** 1 Doctoral School, “Victor Babes” University of Medicine and Pharmacy Timisoara, Eftimie Murgu Square 2, 300041 Timișoara, Romania; amalia.constantinescu@umft.ro; 2 Department of Radiology and Medical Imaging, “Victor Babes” University of Medicine and Pharmacy Timisoara, Eftimie Murgu Square No. 2, 300041 Timișoara, Romania; dmanolescu@umft.ro; 3 Faculty of Medicine, “Victor Babes” University of Medicine and Pharmacy Timisoara, Eftimie Murgu Square No. 2, 300041 Timișoara, Romania; ionel-alin.muntean@student.umft.ro; 4 Department of Computer and Information Technology, Automation and Computers Faculty, “Politehnica” University of Timisoara, Vasile Pârvan Blvd, No. 2, 300223 Timișoara, Romania; versavia.ancusa@upt.ro; 5 Clinical Hospital of Infectious Diseases and Pneumophthisiology “Dr. Victor Babeș”, Gheorghe Adam No. 13, 300226 Timișoara, Romania; alin.nicola@umft.ro; 6 Center for Research and Innovation in Precision Medicine of Respiratory Diseases (CRIPMRD), “Victor Babes” University of Medicine and Pharmacy Timisoara, 300041 Timișoara, Romania; oancea@umft.ro; 7 Department of Pulmonology, “Victor Babes” University of Medicine and Pharmacy Timisoara, 300041 Timișoara, Romania

**Keywords:** CT-guided lung biopsy, needle–pleural angle, pneumothorax, pulmonary hemorrhage, risk stratification, transthoracic needle biopsy

## Abstract

**Background/Objectives:** CT-guided transthoracic core needle biopsy (CT-TTNB) is the standard technique for histological characterization of pulmonary lesions, yet it carries a 15–42% rate of pneumothorax and a 5–27% rate of alveolar hemorrhage. Accurate identification of modifiable and non-modifiable procedural determinants is essential for pre-procedural risk stratification, technique optimization, and post-procedural care. **Methods:** We conducted a single-centre retrospective cohort study of 240 consecutive CT-TTNB procedures performed between November 2023 and January 2025. The variables were extracted from medical records and PACS and entered into univariate and multivariate analysis models. Model performance was assessed by AUC-ROC, Nagelkerke R^2^, and the Hosmer–Lemeshow test. **Results:** Pneumothorax occurred in 79 patients (32.9%), with chest tube drainage required in 14 (5.8%). Alveolar hemorrhage was identified in 49 patients (20.4%). Four independent predictors for pneumothorax were identified: needle–pleural angle (OR 0.837/1°; *p* < 0.001), pleura-to-lesion distance (OR 1.675/10 mm; *p* < 0.001), fissure traversal (OR 26.718; *p* = 0.003), and patient age (OR 1.076/year; *p* = 0.002); AUC-ROC 0.927. The alveolar hemorrhage model identified pleura-to-lesion distance (OR 1.768/10 mm; *p* < 0.001), RUL apical segment (OR 4.281; *p* < 0.001), and carcinomatous lymphangitis as protective factors (OR 0.359; *p* = 0.030); AUC-ROC 0.822. The needle–pleural angle was not independently associated with alveolar hemorrhage, confirming mechanistically distinct complication pathways. **Conclusions:** Needle–pleural angle is the dominant modifiable determinant of pneumothorax and pneumothorax-related complications. Optimizing the trajectory towards perpendicularity does not increase hemorrhage risk. These findings support the implementation of angle-based trajectory planning protocols and risk-stratified post-procedural monitoring.

## 1. Introduction

According to the GLOBOCAN 2022 cancer estimates, lung cancer accounts for 12.4% of all new cancer diagnoses and 18.7% of all cancer-related deaths across both sexes [1]. Early and precise tissue diagnosis is essential for guiding treatment decisions, enabling accurate histological classification, staging, molecular profiling, and assessment of eligibility for targeted therapies and immunotherapy [2].

CT-guided transthoracic core needle biopsy (CT-TTNB) is considered the reference technique for diagnosing pulmonary nodules and masses [3,4]. The diagnostic yield of CT-TTNB ranges from 85 to 95%, with sensitivity for detecting malignancies of 92–97% [5,6]. Although CT-TTNB is highly accurate for evaluating malignant lesions, it is associated with a 30–32% overall complication rate [7,8].

Prior studies show pneumothorax (PNX) as the most frequent complication, with an incidence of 15–42% [3,6,7], requiring chest tube placement in 1.6–7% of cases [5,9,10,11]. A higher risk of PNX is associated with emphysematous lungs, smaller lesions, deeper lesions, multiple pleural punctures, needle gauge, and the interventional radiologist’s experience level [6,9,10].

Pulmonary hemorrhage is the second-most common complication, occurring in 5–18% of cases [5,8], while hemoptysis occurs in 4–5% [9,12]. Risk factors for bleeding include smaller lesions, deep-seated lesions, lesion composition, the number of pleural passes, smoking history, emphysema, and needle gauge [9]. A rare but potentially fatal complication is air embolism, reported in association with subsolid nodules and lower lobe lesions [13].

Although most complications are mild and self-limiting, a non-negligible subset requires immediate intervention, prolonged observation, or hospitalization [14]. Therefore, it is clinically relevant to identify patient and procedural determinants of these complications, as this may help with pre-procedural risk stratification, biopsy technique optimization, and post-procedural care.

While prior studies have identified several procedural and anatomical determinants of PNX and alveolar hemorrhage, key gaps remain. Furthermore, PNX and alveolar hemorrhage risk factors have rarely been evaluated within a single cohort using comparable multivariate frameworks, leaving unresolved whether the two complications share common determinants or follow mechanistically distinct pathways. In the present single-centre retrospective study, we aimed to map the degree of overlap between PNX and alveolar hemorrhage determinants and derive applicable risk thresholds for procedural planning. Additionally, we reported the diagnostic yield achieved with the non-coaxial single-pass technique at our institution.

## 2. Materials and Methods

### 2.1. Study Design

This single-centre retrospective cohort study was conducted at the Department of Radiology, Victor Babes Clinical Hospital, Timisoara, Romania. This study was approved by the institutional Ethics Committee. Before the procedure, patients were informed about the steps and associated risks of TTNB and signed a consent form indicating their understanding.

Consecutive patients who underwent CT-TTNB between November 2023 and January 2025 were considered for inclusion. Inclusion criteria were: (1) age ≥18 years; (2) pulmonary lesions requiring histopathological characterization by CT-guided transthoracic biopsy; (3) successful initiation of the biopsy procedure; and (4) availability of complete pre-procedural clinical assessment, procedural data, and post-procedural imaging follow-up. Exclusion criteria were: (1) target lesions outside the pulmonary parenchyma; (2) incomplete procedural or clinical data; (3) severe pulmonary hypertension; (4) contralateral pneumonectomy, and (5) uncorrected coagulation abnormalities or bleeding disorders contraindicating biopsy. Previous lung interventions were not considered an exclusion criterion if CT-TTNB remained clinically indicated and technically feasible. Patients receiving anticoagulant, antiplatelet, or antithrombotic therapy were included only after appropriate peri-procedural management according to institutional protocols. The final cohort consisted of 240 patients.

### 2.2. Procedure Technique

The biopsy protocol was standardized throughout the study period. All procedures were performed by a single interventional radiologist using a GE Optima CT520 multidetector CT scanner (GE HealthCare, Chicago, IL, USA). Pre-procedural management included clinical and laboratory assessment of each patient. Measurements of platelet count and International Normalized Ratio (INR) are recommended according to the 2019 Society of Interventional Radiology (SIR) guidelines [15]; meanwhile, the 2021 Cardiovascular and Interventional Radiological Society of Europe (CIRSE) guidelines [16] advise assessing prior bleeding history by using structured questionnaires.

All enrolled patients underwent a thorough review of their medical history for previous coagulopathy. Among patients on anticoagulants, antiplatelet agents, or antithrombotic therapy, the medication was withheld and reinitiated according to the current recommendations [15,16].

Pre-procedural CT scans were reviewed to determine lesion characteristics and the optimal needle insertion path. The patient’s position was determined on a case-by-case basis, taking into consideration: lesion location, the length of the needle path, important anatomical structures, and the presence of emphysema or other lung parenchymal pathologies. Respiratory motion was managed through a standardized breath-hold protocol: patients were instructed to hold their breath at end-expiration during each CT acquisition and needle advancement, ensuring reproducibility of lesion position between planning and targeting scans.

Biopsies were performed under local anesthesia (10–20 mL of 2% lidocaine). The position of the biopsy groove varied depending on the lesion morphology. All procedures were performed using a non-coaxial single-pass core biopsy technique with 14-, 16-, or 18-gauge semi-automatic Tru-Cut needles, which represented the standard institutional approach during the study period. The use of this technique reflected institutional workflow and equipment availability rather than a deliberate preference over coaxial systems [9,17].

A critical component for ensuring patient safety was post-procedural imaging. A low-dose CT scan was performed immediately after needle removal to identify possible complications, such as PNX, hemorrhage, or air embolism. Patients were observed for 2 h post-procedure for any clinical deterioration, and a chest X-ray was performed routinely to screen for subclinical complications.

### 2.3. Variables and Definitions

All data were retrospectively extracted from institutional picture archiving and communication systems (PACS) and medical records by 2 trained reviewers. The recorded variables were organized into four categories: patient-related variables, lesion-related variables, procedural variables, and complications.

#### 2.3.1. Patient-Related Variables

The following patient-related variables were recorded: age (years), sex, body mass index (kg/m^2^), smoking history (pack–years), occupational exposure, comorbidities, and coagulation parameters (APTT and INR).

Occupational exposure was recorded as a binary categorical variable (present/absent) and further specified by exposure type (paint fumes, shoe polish, chemical solvents, and other inhaled substances potentially associated with pulmonary pathology), where applicable.

Based on the patient’s medical record before the procedure, the comorbidities included were chronic obstructive pulmonary disease (COPD), tuberculosis sequelae, cardiovascular disease, and diabetes mellitus, among others. All clinically significant conditions were documented regardless of organ system or prevalence within the cohort.

#### 2.3.2. Lesion-Related Variables

Lesion characteristics were evaluated on pre-procedural CT imaging by a team of 2 dedicated radiologists. The following variables were recorded: lesion location, lesion type, and dimensions. Nodules were further categorized as solid, subsolid (part-solid), or ground-glass opacity [18] and were assessed for morphological characteristics, including spiculation, central necrosis, and cavitation. The maximum lesion diameter was analyzed as a continuous variable. Parenchymal and pleural findings were also documented as binary variables: emphysema, pulmonary fibrosis, carcinomatous lymphangitis, bronchiectasis, and pleural effusion.

#### 2.3.3. Procedural Variables

**Figure 1:**
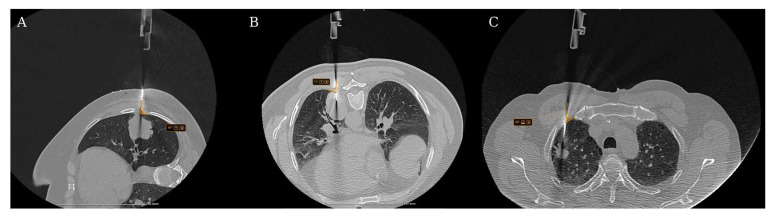
Intraprocedural CT images illustrating needle–pleural angle measurement: (**A**) 90°—perpendicular trajectory (lateral approach), (**B**) 71°—moderately oblique trajectory (posterior approach), (**C**) 49°—markedly oblique trajectory (anterior approach).

The following procedural variables were recorded for each biopsy: needle gauge (14 G, 16 G, 18 G), needle length (mm), needle insertion angle (measured as the acute angle formed between the needle trajectory and the tangent to the pleural surface at the puncture site (Figure 1)), and the number of biopsy fragments obtained per procedure.

Based on the patient’s positioning and the needle insertion path, the puncture approach was recorded as anterior, posterior, or lateral. The pleural-to-nodule distance was defined as the shortest distance from the pleural surface to the nearest margin of the target lesion. Pleura-to-lesion distance was evaluated as a continuous variable. Interobserver reliability was excellent for needle–pleura angle (ICC = 0.983, 95% CI: 0.971–0.990) and pleura-to-lesion distance (ICC = 0.996, 95% CI: 0.993–0.997). All measurements were obtained independently by two trained reviewers, with each blinded to the other’s values; complete outcome blinding was precluded by the retrospective design. For both the needle–pleura angle and the pleura-to-lesion distance, clinically meaningful binary thresholds were determined post hoc by maximizing the Youden index in ROC curve analysis against the respective primary outcomes. The resulting thresholds (75° for needle-to-pleura angle and 22 mm for pleura-to-lesion distance) were applied for descriptive risk stratification and are reported in the Results section.

#### 2.3.4. Histopathological Results

Histopathological results were recorded for all patients and classified as malignant, benign, non-diagnostic, or not obtained. For malignant cases, histological subtype, immunohistochemical profile, and the presence of inflammatory reaction were documented. Cases in which the initial biopsy was non-diagnostic and a repeat biopsy was performed were recorded as a binary variable.

### 2.4. Complications

Complications were diagnosed based on clinical assessment and post-procedural CT imaging and were graded into five severity tiers: mild, moderate, severe, life-threatening, and fatal [14].

PNX was defined as any new air accumulation in the pleural space identified on immediate post-procedural CT. PNX rim thickness was measured at two time points: immediately after needle withdrawal and ten minutes later to assess for progression. PNX managed conservatively was classified as mild, PNX that required needle aspiration as moderate, and PNX that required chest tube placement as severe.

Pulmonary hemorrhage was defined as any new ground-glass opacity or consolidation along the needle tract identified on post-procedural CT. Hemoptysis was defined as any expectoration of blood occurring during or within 24 h of the procedure. Both complications were classified as mild if the patient did not require medical intervention, moderate if the patient required pharmacological treatment or bronchoscopic evaluation, or severe if the patient required invasive intervention.

Hemothorax was defined as any new fluid accumulation in the pleural space, with CT attenuation values greater than 15.6 Hounsfield units (HU) detected on post-procedural imaging. The cases that required only monitoring were classified as mild. Hemothorax that required thoracocentesis was classified as moderate, and the cases that necessitated surgical intervention or transarterial embolization were classified as severe.

### 2.5. Outcomes

Primary outcomes were the occurrence of PNX and pulmonary hemorrhage following CT-TTNB, each analyzed as an independent binary endpoint. These outcomes were analyzed separately, given their partially overlapping but distinct risk factor profiles.

The secondary outcomes were: (1) PNX requiring chest tube drainage; (2) hemoptysis occurrence; (3) hemothorax occurrence; and (4) diagnostic yield.

Diagnostic yield was defined as the proportion of procedures yielding a specific histopathological diagnosis out of the total number of procedures performed.

### 2.6. Statistical Analysis

All statistical analyses were performed using MedCalc Statistical Software (version 23.4.9; MedCalc Software Ltd., Ostend, Belgium). A two-tailed *p*-value < 0.05 was considered statistically significant for all analyses.

The variables were analyzed descriptively for normality using the Shapiro–Wilk test. Means ± standard deviations (SDs) were used for normally distributed variables, while non-normally distributed variables were expressed as median with interquartile range (IQR). Categorical variables were reported as absolute frequencies and percentages.

To identify potential determinants, univariate analyses were performed separately for each primary outcome. Statistically significant variables (*p* < 0.10) were subsequently entered into a binary logistic regression model. Multicollinearity among candidate variables was assessed, and, where necessary, only the clinically most relevant variable was retained. The results were expressed as odds ratios (ORs) with corresponding 95% confidence intervals (CIs). For continuous predictors that demonstrated a clinically relevant dose–response correlation, odds ratios were scaled per 10 mm increase in order to enhance clinical interpretability. The goodness of fit of each model was assessed using the Hosmer–Lemeshow test. Model explanatory power was reported using the Nagelkerke R^2^ statistic.

The discriminative ability of each logistic regression model was assessed by receiver operating characteristic (ROC) curve analysis. The area under the ROC curve (AUC) was calculated separately for each logistic regression model. Acceptable discrimination was indicated by AUC values of 0.70–0.79, good discrimination by AUC values of 0.80–0.89, and excellent discrimination by AUC values ≥ 0.90. AUC values are presented with 95% confidence intervals. The optimal cutoff point was determined by maximizing the Youden index.

To assess internal validity and quantify optimism, bootstrap resampling (1000 iterations) was applied to both primary logistic regression models. Bootstrap-corrected AUC values and threshold stability were reported alongside the original estimates.

Decision curve analysis was performed to evaluate the net clinical benefit of each logistic regression model across a range of threshold probabilities, using the dcurves package (version 0.5.0) in R statistical software (version 4.6.0, R Foundation for Statistical Computing, Vienna, Austria).

Data completeness was ensured through comprehensive review of institutional PACS and medical records. All cases included in the final analysis had complete data for all recorded variables.

## 3. Results

### 3.1. Study Population and Diagnostic Yield

A total of 240 CT-guided percutaneous transthoracic needle biopsies (PTNBs) were included in the final analysis. The median patient age was 67 years (IQR 58–73), 68.8% were male, and 31.2% were female. Smoking history was documented in 179 patients (74.6%), including 125 current smokers (52.1%), 54 former smokers (22.5%), and 61 patients who never smoked (25.4%). The demographic profile of our cohort is consistent with the regional lung cancer population described in prior studies [19].

The distribution of lesions was 59.2% in the right lung, 36.2% in the left lung, and 3.8% bilaterally, with the right upper lobe (RUL), right lower lobe (RLL), and left upper lobe (LUL) representing the most frequent locations. The median maximum lesion diameter was 46 mm (IQR 28–66 mm). The median pleural-to-lesion distance was 12 mm (IQR 0–25 mm). Co-existing lesions, such as emphysema, carcinomatous lymphangitis, and pulmonary effusion, were present in 48.8%, 30.4%, and 18.3% of cases, respectively.

Procedures were performed via a posterior approach in 119 cases (49.6%), a lateral approach in 64 cases (26.7%), and an anterior approach in 57 cases (23.8%). Fissure traversal occurred in 19 cases (7.9%). The median needle–pleural angle was 80.5° (IQR 69–87°); an angle below 75° was recorded in 93 procedures (38.75%).

Histopathological confirmation of malignancy was achieved in 204 of 240 procedures (85%), including two additional malignant diagnoses obtained after repeated biopsy. The histological profile of our cohort is consistent with institutional data reported previously [20]. Overall, 36 of 240 cases (15%) were either non-malignant or non-confirmatory. Among these, 14 cases (5.8%) were non-diagnostic due to insufficient viable tissue or extensive necrosis that prevented definitive histopathological interpretation; 11 cases (4.6%) exhibited inflammatory changes, including three consistent with tuberculosis; and in 11 patients (4.6%), no specimen was acquired due to premature procedure termination caused by early PNX or patient intolerance. When considering only cases in which a specimen was successfully acquired (*n* = 229), the adjusted diagnostic yield for malignancy increased to 89.1%.

### 3.2. Incidence of Complications

At least one complication was recorded in 111 patients (46.2%), while 129 patients (53.8%) underwent the procedure without any adverse events. The most frequent complication was PNX, occurring in 79 patients (32.9%), of whom 65 (27.1% of the total cohort) were managed conservatively, while 14 (5.8% of the total cohort) required chest tube drainage. Alveolar hemorrhage was identified in 49 patients (20.4%) and hemoptysis in 13 patients (5.4%). Hemothorax was rare, occurring in three patients (1.2%). All hemorrhagic complications were managed conservatively. The distribution of post-procedural complications according to severity and management is summarized in Table 1.

**Table 1:** Incidence, severity, and management of post-procedural complications.

Complication	*n* (%)	Clinical Severity (SIR-Based)	Management
Pneumothorax—conservative	65 (27.1)	Mild	Clinical observation
Pneumothorax requiring drainage	14 (5.8)	Moderate	Chest tube drainage
Alveolar hemorrhage	49 (20.4)	Mild	Conservative
Hemoptysis	13 (5.4)	Mild	Conservative
Hemothorax	3 (1.2)	Mild	Conservative

### 3.3. Determinants of PNX

**Figure 2:**
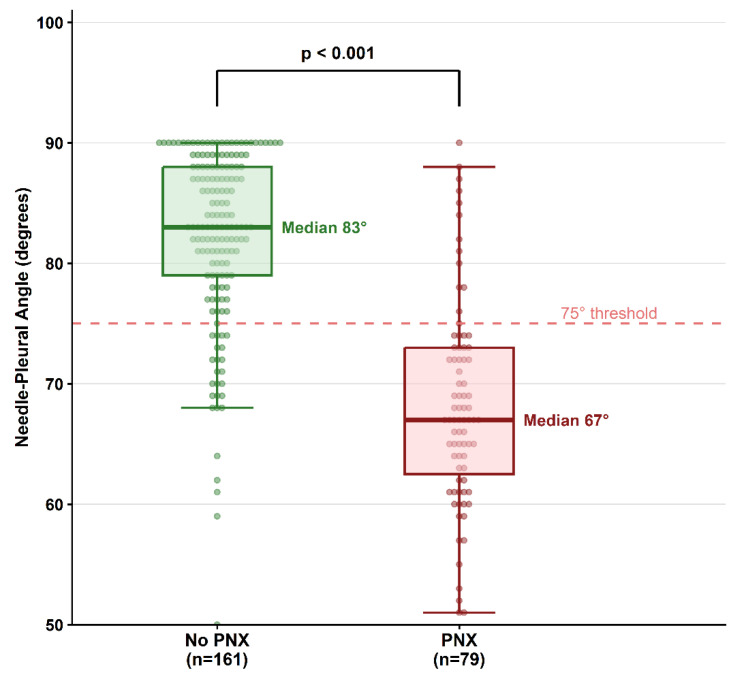
Needle–pleural angle by pneumothorax status.

Univariate analysis revealed several procedural, anatomical, and patient-level factors as significant predictors (Table 2). The dominant predictor was the needle–pleural angle: patients who developed PNX had a substantially lower median angle compared to those without PNX (67.0°, IQR 62.5–73.0° vs. 83.0°, IQR 79.0–88.0°; *p* < 0.001). When dichotomized at 75°, a needle angle below this threshold was associated with a markedly elevated risk of PNX (OR 28.99, 95% CI 13.77–61.01; *p* < 0.001) (Figure 2).

**Table 2:** Patient, lesion, and procedural characteristics stratified by PNX occurrence (*n* = 240).

Characteristics	No PNX (*n* = 161)	PNX (*n* = 79)	*p*-Value
***Patient characteristics***			
Age (years), median [IQR]	67 [56–72]	69 [63–74]	**0.019**
Sex—male, *n* (%)	107 (66.5%)	58 (73.4%)	0.302
BMI (kg/m^2^), median [IQR]	26.7 [23.3–29.3]	25.5 [23.0–29.4]	0.641
Smoking status, *n* (%)			
Ever smoked	121 (75.2%)	58 (73.4%)	0.755
Pack–years (ever-smokers), median [IQR]	30 [0–40]	30 [0–40]	0.733
Modified coagulation times (%)	29.2%	31.9%	0.665
INR, median [IQR]	1.0 [0.9–1.1]	1.0 [0.9–1.1]	0.649
***Lesion characteristics***			
Maximum diameter (mm), median [IQR]	49 [28–73.5]	39.5 [28.2–56]	**0.027**
Pleura-to-lesion distance (mm), median [IQR]	0 [0–22]	22 [12–33.5]	**<0.001**
Lesion morphology, *n* (%)			
Solid nodule	140 (87.0%)	68 (86.1%)	0.959
Spiculation	119 (73.9%)	65 (85.5%)	0.109
Central necrosis	51 (33.1%)	28 (33.3%)	1.000
Parenchymal background, *n* (%)			
Emphysema	72 (44.7%)	45 (57.0%)	0.074
Carcinomatous lymphangitis	61 (36.7%)	18 (24.7%)	0.074
Pleural effusion	75 (38.3%)	4 (9.1%)	**<0.001**
***Procedural characteristics***			
Biopsy approach, *n* (%)			
Posterior	81 (50.3%)	38 (48.1%)	0.784
Lateral	36 (22.4%)	28 (35.4%)	**0.043**
Anterior	44 (27.3%)	13 (16.5%)	0.076
Needle–pleural angle (°), median [IQR]	83 [79–88]	67 [63–73]	**<0.001**
Needle–pleural angle < 75°, *n* (%)	26 (16.1%)	67 (84.8%)	**<0.001**
Needle gauge, *n* (%)			
14 gauge	9 (5.6%)	3 (3.8%)	0.755
16 gauge	71 (44.1%)	31 (39.2%)	0.491
18 gauge	81 (50.3%)	45 (57.0%)	0.340
Needle length (mm), median [IQR]	120 [100–120]	120 [100–120]	0.344
Fissure traversal, *n* (%)	61 (27.7%)	17 (89.5%)	**<0.001**
DLP (mGy·cm^2^), median [IQR]	788 [502–1304]	1003 [537–1344]	0.269

Fissure traversal was significantly associated with PNX, with an odds ratio of 22.16 (95% CI 4.97–98.76; *p* < 0.001). PNX was observed in 89.5% of patients with documented fissure traversal, compared to only 27.7% of those without it. The pleura-to-lesion distance was greater in the PNX group (median 22.0 mm vs. 0.0 mm, *p* < 0.001). Pleural effusion exerted a strong protective effect (9.1% vs. 38.3%; OR 0.16, *p* < 0.001), consistent with a fluid tamponade mechanism, while maximum lesion diameter was inversely associated with PNX (*p* = 0.027). A lateral approach was associated with a higher PNX rate compared to anterior or posterior approaches (*p* = 0.043). Patient age was modestly higher in the PNX group (median 69.0 vs. 67.0 years; *p* = 0.019). Emphysema was present in 38.5% of PNX patients vs. 27.3% without (OR 1.67, *p* = 0.074); while not statistically significant, the direction and magnitude are clinically meaningful. Carcinomatous lymphangitis showed a lower PNX rate (24.7% vs. 36.7%; OR 0.56, *p* = 0.074).

Variables with univariate *p* < 0.10 or clinical importance were entered into sequential models. Emphysema (borderline univariate *p* = 0.074) and lesion size were tested in intermediate models and removed upon demonstrating non-significance after adjustment. The final multivariate model identified four independent determinants: needle–pleural angle, pleura-to-lesion distance, fissure traversal, and patient age (Table 3).

**Table 3:** PNX final multivariate logistic regression models.

Variable	OR	95% CI	*p*-Value
***PNX (n = 240; events = 79; AUC = 0.927; Nagelkerke R^2^ = 0.656)***			
Needle–pleural angle (per 1° increase)	0.837	0.795–0.881	**<0.001**
Pleura-to-lesion distance (per 10 mm)	1.675	1.260–2.226	**<0.001**
Fissure traversal	26.718	3.074–232.185	**0.003**
Patient age (per 1 year)	1.076	1.026–1.128	**0.002**
Pleural effusion	0.385	0.122–1.206	0.101

The needle–pleural angle was the dominant modifiable predictor (OR 0.837 per 1° increase, 95% CI 0.795–0.881; *p* < 0.001): each additional degree of angle towards perpendicularity was associated with a 16.3% reduction in the odds of PNX. Pleura-to-lesion distance independently increased risk (OR 1.675 per 10 mm, 95% CI 1.260–2.226; *p* < 0.001).

Fissure traversal significantly increased the odds of PNX (OR 26.718, 95% CI 3.074–232.185; *p* = 0.003). However, the wide confidence interval reflects the limited number of cases of fissure traversal (*n* = 19). Patient age was an independent predictor (OR 1.076 per year, 95% CI 1.026–1.128; *p* = 0.002).

Pleural effusion was retained in the final model as a likely protective factor (OR 0.385, 95% CI 0.122–1.206; *p* = 0.101), despite not reaching adjusted significance, in view of its large univariate odds ratio (OR 0.16) and strong biological rationale; a sensitivity model excluding pleural effusion yielded near-identical discrimination (AUC 0.923 vs. 0.927), with the four remaining predictors retaining full significance.

Lateral approach, emphysema, and lesion size were not independent predictors after adjustment, confirming that their univariate associations are fully explained by the trajectory variables.

Model performance was outstanding. The AUC-ROC was 0.927, indicating excellent discriminative ability. Calibration was excellent, with a Hosmer–Lemeshow χ^2^ of 3.84 (df = 8, *p* = 0.871). The Nagelkerke pseudo-R^2^ was 0.656. At the Youden-optimal threshold (p* = 0.723), sensitivity was 83.5%, specificity 88.8%, PPV 78.6%, and NPV 91.7%, with 66 of 79 PNX events correctly identified (true positives) and 143 of 161 non-PNX cases accurately classified (true negatives). Bootstrap internal validation yielded an optimism-corrected AUC of 0.918, confirming minimal overfitting. The Youden-optimal threshold of 75° for needle–pleural angle was stable across bootstrap samples (bootstrap mean 75.4°, 95% CI 73.5–78.5°).

Decision curve analysis demonstrated that the pneumothorax model provided net clinical benefit over both the ‘treat-all’ and ‘treat-none’ strategies across the entire range of clinically relevant threshold probabilities (5–85%), confirming its practical utility for pre-procedural risk stratification (Supplementary Figure S1, Panel A).

### 3.4. PNX Rim Progression (Explanatory, EPV = 7.5)

Among the 79 patients who developed PNX, rim progression was observed in 19.5% of PNX patients, while 80.5% were stable or showed a decrease in rim size at the 10 min reevaluation. The median initial rim was 10 mm (IQR 7–17 mm), and the median 10 min rim was 10 mm (IQR 7–19 mm).

Univariate analysis identified the needle–pleural angle as the strongest predictor of rim progression (*p* = 0.002). Fissure traversal was also significantly associated with progression (OR 5.06, 95% CI 1.47–17.41; *p* = 0.012). In the final model, both the needle–pleural angle (OR 0.898 per 1°, 95% CI 0.818–0.984; *p* = 0.022) and fissure traversal (OR 3.753, 95% CI 1.001–14.075; *p* = 0.050) were independently associated with rim progression. The model achieved an AUC-ROC of 0.789, a Nagelkerke R^2^ = 0.249, and adequate calibration (Hosmer–Lemeshow *p* = 0.662) .

### 3.5. PNX Requiring Chest Tube Drainage (Explanatory, EPV = 7)

Chest tube drainage was required in 14 patients (5.8% of the total cohort; 17.7% of patients with PNX). Univariate analysis identified a cluster of procedural and anatomical predictors. The needle–pleural angle was the most powerful predictor: patients requiring drainage had a substantially lower median angle (59.5°, IQR 55.0–64.0°) compared to those who did not (81.0°, IQR 72.0–87.0°; *p* < 0.001). A needle angle below 75° was universally present among drainage patients (*p* < 0.001). Fissure traversal was strongly associated with drainage (OR 12.28, 95% CI 3.71–40.69; *p* < 0.001). A lateral biopsy approach conferred a significantly higher risk compared to both anterior (OR 5.56, 95% CI 1.79–17.30; *p* = 0.003) and posterior approaches (OR 6.32, 95% CI 1.64–24.29; *p* = 0.005). Pleura-to-lesion distance was significantly greater in the drainage group (median 26.0 mm, IQR 22.0–38.0 mm; *p* < 0.001). Although the 18 G needle was associated with increased odds of chest tube drainage in univariate analysis (OR 13.12, 95% CI 1.69–101.96; *p* = 0.002), this association was no longer significant after adjustment for needle–pleural angle and biopsy approach (OR 5.566; *p* = 0.203). Given the smaller diameter of 18 G needles compared with 14 G/16 G systems, this finding likely reflects confounding by procedural characteristics rather than a direct effect of needle size. Among location variables, the superior lingula segment was associated with a markedly elevated drainage rate (OR 30.54, 95% CI 4.62–201.95; *p* < 0.001), as was the RLL anterior basal segment (OR 9.25, 95% CI 1.53–55.62; *p* = 0.015). Carcinomatous lymphangitis was associated with complete protection against chest tube drainage; none of the 73 patients with lymphangitis required drainage, compared to 14 of 165 patients without lymphangitis (8.5%; Fisher’s exact *p* = 0.006).

Given the event count of 14, multivariate modelling was restricted to at most two predictors (EPV = 7.0), and all results are therefore exploratory. Carcinomatous lymphangitis was excluded due to complete separation (0 drainage events among 73 lymphangitis patients), which prevents logistic regression convergence. Sparse segmental location variables were excluded due to insufficient cell counts for stable estimation. The final model retained two independent predictors: needle–pleural angle (OR 0.723 per 1°, 95% CI 0.621–0.843; *p* < 0.001) and lateral approach (OR 17.824, 95% CI 2.845–111.680; *p* = 0.002). Despite the EPV constraint, the model demonstrated exceptional discrimination (AUC-ROC = 0.979), a Nagelkerke R^2^ of 0.639, and outstanding calibration (Hosmer–Lemeshow *p* = 0.995). At the Youden-optimal threshold (p* = 0.122), sensitivity was 100% and specificity 95.1%, with an NPV of 100%, meaning no drainage event was missed by the model (0 false negatives). These metrics must be interpreted with caution, given the small event count, and prospective validation in a larger cohort is required.

### 3.6. Determinants of Alveolar Hemorrhage

**Figure 3:**
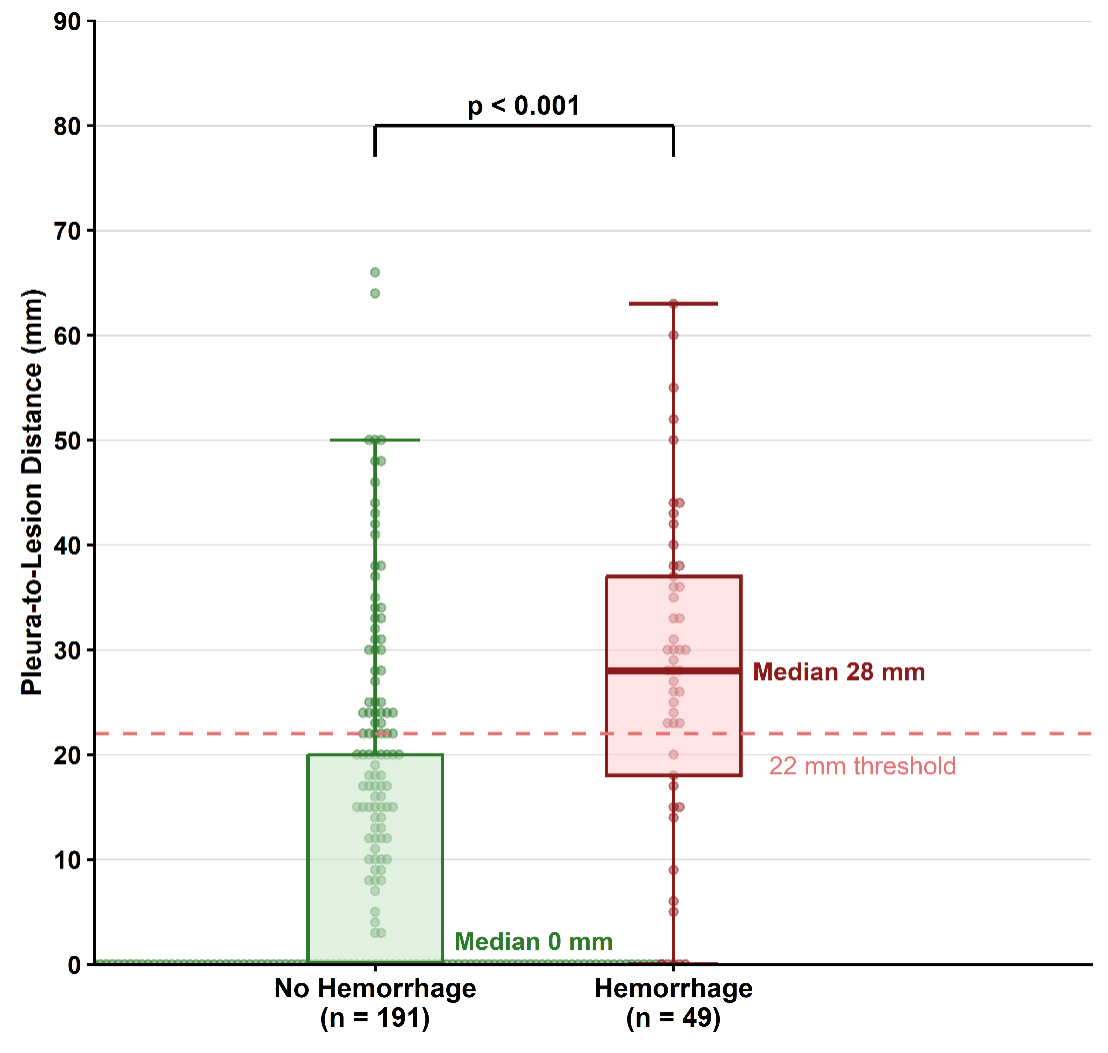
Pleura-to-lesion distance by alveolar hemorrhage status.

Univariate analysis identified seven variables as statistically significant risk factors (Table 4). Patients who developed pulmonary hemorrhage had a greater median pleura-to-lesion distance than patients without hemorrhage (28 mm, IQR 18–37 mm vs. 0.0 mm, IQR 0–20 mm, *p* < 0.001). When dichotomized at 22 mm, a pleura-to-lesion distance greater than this threshold was associated with an elevated risk of alveolar hemorrhage (OR 9.437, 95% CI 4.63–19.21; *p* < 0.001) (Figure 3). The maximum lesion diameter was inversely correlated with hemorrhage (*p* = 0.018), with smaller lesions more frequently affected (median 36 mm in the hemorrhage group vs. 49 mm in the non-hemorrhage group). Carcinomatous lymphangitis was associated with a reduced risk of hemorrhage (OR 0.44, 95% CI 0.20–0.97; *p* = 0.039). Lesion location in the right lung was associated with an increased hemorrhage rate (OR 2.22, 95% CI 1.11–4.45; *p* = 0.023), with the RUL apical segment demonstrating the strongest location-specific association (OR 3.39, 95% CI 1.60–7.19; *p* = 0.003). Among procedural factors, needle length was significantly longer in the hemorrhage group (*p* < 0.001). Pack–year burden also reached significance (*p* = 0.044), although the median values were nearly identical between groups (30 pack–years in both). Variables not associated with alveolar hemorrhage included: age, sex, BMI, smoking status, lesion morphology, biopsy approach, needle gauge, needle–pleural angle, fissure traversal, number of core samples, and PNX.

**Table 4:** Patient, lesion, and procedural characteristics stratified by alveolar hemorrhage occurrence (*n* = 240).

Characteristics	No Hemorrhage (*n* = 191)	Hemorrhage (*n* = 49)	*p*-Value
***Patient characteristics***			
Age (years), median [IQR]	67 [59–72]	67 [56–74]	0.669
Sex—male, *n* (%)	129 (67.5%)	36 (73.5%)	0.492
BMI (kg/m^2^), median [IQR]	26.7 [23.3–29.5]	24.8 [22.5–28.8]	0.288
Smoking status, *n* (%)			
Ever smoked	89 (46.6%)	25 (51.0%)	0.140
Pack–years (ever-smokers), median [IQR]	30 [0–40]	30 [20–40]	**0.044**
Modified coagulation times, *n* (%)	61 (31.9%)	10 (20.4%)	0.160
INR, median [IQR]	1.0 [0.9–1.1]	1.0 [0.9–1.1]	0.432
***Lesion characteristics***			
Maximum diameter (mm), median [IQR]	49 [30–70]	36 [26–54]	**0.018**
Pleura-to-lesion distance (mm), median [IQR]	0 [0–20]	28 [18–37]	**<0.001**
Laterality—right lung, *n* (%)	106 (55.5%)	36 (73.5%)	**0.023**
RUL apical segment (S1), *n* (%)	22 (11.5%)	15 (30.6%)	**0.003**
Lesion morphology, *n* (%)			
Solid nodule	164 (85.9%)	44 (89.8%)	0.746
Spiculation	141 (73.8%)	43 (87.8%)	**0.316**
Central necrosis	33 (21.4%)	15 (17.9%)	0.613
Parenchymal background, *n* (%)			
Emphysema	92 (48.2%)	25 (51.0%)	0.873
Carcinomatous lymphangitis	40 (24.1%)	9 (12.3%)	**0.039**
Pleural effusion	44 (22.4%)	5 (11.4%)	0.146
***Procedural characteristics***			
Biopsy approach, *n* (%)			
Posterior	95 (49.7%)	24 (49.0%)	1.000
Lateral	52 (27.2%)	12 (24.5%)	0.856
Anterior	44 (23.0%)	13 (26.5%)	0.579
Needle–pleural angle (°), median [IQR]	81 [69–87]	78 [70–86]	0.777
Needle–pleural angle < 75°, *n* (%)	69 (36.1%)	22 (44.9%)	0.260
Needle gauge, *n* (%)			
14 gauge	10 (5.2%)	2 (4.1%)	1.000
16 gauge	83 (43.5%)	19 (38.8%)	0.628
18 gauge	98 (51.3%)	28 (57.1%)	0.523
Needle length (mm), median [IQR]	120 [100–120]	120 [100–160]	**<0.001**
Fissure traversal, *n* (%)	45 (20.5%)	4 (21.1%)	1.000
DLP (mGy·cm^2^), median [IQR]	802 [494–1259]	993 [589–1541]	0.430

Variables with univariate significance were entered into sequential logistic regression models. The final multivariate model identified four independent determinants of alveolar hemorrhage: pleura-to-lesion distance, carcinomatous lymphangitis, needle length, and RUL apical segment location (Table 5). The strongest predictor was pleura-to-lesion distance (OR 1.768 per 10 mm increase, 95% CI 1.410–2.216; *p* < 0.001). Each additional 10 mm of parenchymal traversal was associated with a 76.8% risk increase in hemorrhage. Carcinomatous lymphangitis emerged as an independent protective factor (OR 0.359, 95% CI 0.142–0.906; *p* = 0.030), while RUL apical segment location conferred a fourfold increase in risk (OR 4.281, 95% CI 1.838–9.974; *p* < 0.001). Despite borderline significance (*p* = 0.06), needle length was retained in the final model, suggesting plausibility as a risk factor independent of pleura-to-lesion distance. Pack–year, maximum lesion diameter, and right lung location did not survive multivariate adjustment.

**Figure 4:**
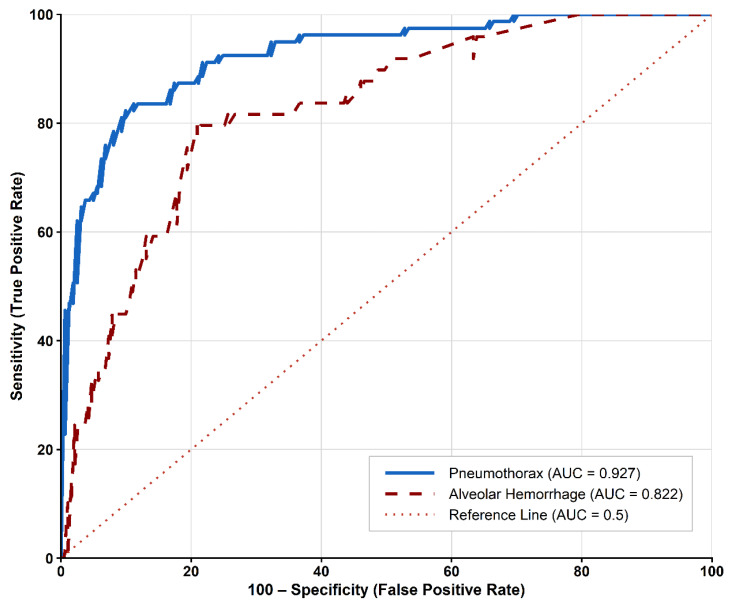
ROC curves—primary outcome prediction models.

Model discrimination was good, with an area under the receiver operating characteristic curve (AUC-ROC) of 0.822 (Figure 4). Model calibration was adequate, as indicated by a non-significant Hosmer–Lemeshow test (χ^2^ = 7.627, df = 8, *p* = 0.470). The Nagelkerke pseudo-R^2^ was 0.319. At the Youden-optimal probability threshold (p* = 0.586), the model achieved a sensitivity of 79.6%, specificity of 79.1%, positive predictive value (PPV) of 49.4%, and negative predictive value (NPV) of 93.8%, correctly identifying 39 of 49 hemorrhage events (true positives) and 151 of 191 non-hemorrhage cases (true negatives) (Table 6). Bootstrap internal validation yielded an optimism-corrected AUC of 0.809, confirming adequate model stability. The Youden-optimal threshold of 22 mm for pleura-to-lesion distance demonstrated acceptable bootstrap stability (bootstrap mean 21.09 mm, 95% CI 4.5–25.5 mm).

**Table 5:** Alveolar hemorrhage final multivariate logistic regression models.

Variable	OR	95% CI	*p*-Value
***Alveolar hemorrhage (n = 240; events = 49; AUC = 0.822; Nagelkerke R^2^ = 0.319)***			
Pleura-to-lesion distance (per 10 mm)	1.768	1.410–2.216	**<0.001**
RUL apical segment (S1)	4.281	1.838–9.974	**<0.001**
Carcinomatous lymphangitis	0.359	0.142–0.906	**0.030**
Needle length (per 10 mm)	1.161	0.993–1.357	0.060

**Table 6:** Summary of model quality metrics—CT-guided transthoracic needle biopsy (*n* = 240).

Outcome	*n*	Events	EPV	R^2^	AUC	HL p	Sens.	Spec.	NPV
Alveolar Hemorrhage	240	49	12.2	0.319	0.822 *	0.470	79.6%	79.1%	93.8%
Pneumothorax	240	79	15.6	0.656	0.927 *	0.871	83.5%	88.8%	91.7%
Rim Progression †	76	15	7.5	0.249	0.789	0.662	80.0%	70.5%	93.5%
Chest Tube Drainage †	240	14	7.0	0.639	0.979	0.995	100%	95.1%	100%

* Bootstrap-corrected AUC: pneumothorax, 0.918; alveolar hemorrhage, 0.809. † Exploratory: EPV < 10. *n* = sample size; EPV = events per variable; R^2^ = Nagelkerke pseudo-R^2^; AUC = area under the ROC curve (Wilcoxon method); HL p = Hosmer–Lemeshow goodness-of-fit *p*-value; Sens. = sensitivity; Spec. = specificity; NPV = negative predictive value. All metrics at the Youden-optimal threshold. Hemoptysis (*n* = 13 events): multivariate analysis not feasible—reported descriptively only.

Decision curve analysis confirmed the clinical utility of the alveolar hemorrhage model, which provided net benefit over both reference strategies across threshold probabilities from 5% to 65% (Supplementary Figure S1, Panel B).

### 3.7. Determinants of Hemoptysis

Hemoptysis occurred in 13 patients (5.4%). Owing to the very low event count, multivariate analysis was not feasible, and results are presented as descriptive and hypothesis-generating observations only. In the univariate analysis, only two variables were statistically significant: alveolar hemorrhage and RML lateral segment location. Alveolar hemorrhage was strongly associated with hemoptysis (OR 16.07, 95% CI 4.23–61.09; *p* < 0.001), a pattern consistent with hemoptysis representing the central airway expression of the same vascular injury rather than an independent complication. The RML lateral segment was also significantly associated with hemoptysis (OR 6.51, 95% CI 1.55–27.41; *p* = 0.026), though this association is based on only three events among 13 segment-level cases and cannot be confirmed as independent given the sample size constraints. All other patient, lesion, and procedural variables were non-significant on univariate analysis (all *p* ≥ 0.05). A larger prospective series is required to characterize the independent predictors of this complication.

### 3.8. Hemothorax

Hemothorax was identified in three patients (1.2%). All three cases were classified as mild and managed conservatively, without requiring thoracocentesis or surgical intervention. The event count was insufficient for meaningful univariate or multivariate analysis, and this outcome is therefore described narratively only.

## 4. Discussion

### 4.1. Main Findings

In this single-centre retrospective cohort of 240 CT-guided transthoracic core needle biopsies, we identified distinct but partially overlapping independent determinants for the two primary complications. For PNX, the needle–pleural angle emerged as the dominant modifiable predictor, as each one-degree increase towards perpendicularity was associated with a 16.3% reduction in PNX odds (OR 0.837; *p* < 0.001), and the overall model achieved outstanding discrimination (AUC-ROC 0.927) with excellent calibration (Hosmer–Lemeshow *p* = 0.871). Pleura-to-lesion distance, interlobar fissure traversal, and patient age independently amplified risk. For alveolar hemorrhage, pleura-to-lesion distance was again the dominant anatomical driver (OR 1.768 per 10 mm; *p* < 0.001), while RUL apical segment location independently quadrupled risk and carcinomatous lymphangitis conferred significant protection—neither of which has been consistently reported in the prior literature. Importantly, the needle–pleural angle was not independently associated with alveolar hemorrhage, confirming that the two complications share anatomical though not technical determinants, a distinction with direct implications for procedural planning.

Secondary analyses further demonstrated the dominant role of needle trajectory. The chest tube drainage model (AUC 0.979) and the PNX rim progression model (AUC 0.789) both identified an oblique needle angle as the principal predictor, along with lateral approach and fissure traversal, respectively. Though constrained by low event counts (EPV < 10) and therefore classified as exploratory, these analyses provide a coherent picture: needles directed at near-perpendicular angles to the pleural surface generate smaller, more easily self-sealing pleural defects, traverse shorter intrapulmonary paths, and are less likely to cross fissural planes, therefore reducing risk at every stage of the complication cascade.

### 4.2. Comparison with the Existing Literature

#### 4.2.1. PNX: Incidence and Risk Factors

The observed PNX incidence of 32.9% is at the upper end of reported ranges (15–42%) [7,9,10,12]. This finding is most plausibly explained by our systematic use of immediate post-procedural low-dose CT, as 65 of 79 PNX cases (82.3%) were managed conservatively without intervention, suggesting most were clinically mild events captured only because of sensitive CT surveillance. Our chest tube drainage rate of 5.8% falls within the meta-analytically derived pooled estimate of 6.9% (95% CI 5.2–9.1%) reported by Huo et al. [10] and the 1.6–7% range across individual series [5,9,10,11], confirming that the severity distribution of our complications is typical despite the higher overall incidence.

The central role of the needle–pleural angle aligns with emerging institutional evidence. Huo et al. [10] identified needle angle as a significant predictor of PNX, while more recent evidence from Peng et al. [21] demonstrated that pleural puncture angle represents an independent predictor of immediate PNX after CT-guided lung biopsy, further supporting the relevance of needle–pleural interaction during procedural planning. Our continuous OR estimate (0.837 per degree) extends this finding by providing a clinically actionable quantitative gradient: operators can now translate a planned angle into a predicted risk reduction. The strong binary effect at the 75° threshold—patients with angles below 75° were 28-fold more likely to develop PNX on univariate analysis—is consistent with institutional experience suggesting that oblique trajectories disrupt larger areas of the visceral pleura and create longer, more irregularly shaped needle tracts that seal less efficiently. These findings echo the institutional learning curve described by Johansen et al. [22], who demonstrated that protocol-driven optimization of needle approach angles was temporally associated with reductions in both PNX incidence and chest tube placement rates at a high-volume centre.

Fissure traversal (OR 26.718) carries a wide confidence interval (3.074–232.185) reflecting the small number of events (*n* = 19), but its biological plausibility is well-supported. Our point estimate substantially exceeds the OR of approximately 3.7 for fissure crossing reported in meta-analysis [10], likely because our non-coaxial technique offers no protective outer sheath during fissure transit—every crossing represents a direct, unguarded pleural puncture. The strong collinearity between fissure traversal and lesion depth (median pleura-to-lesion distance: 33 mm vs. 0 mm in non-traversal cases; *p* < 0.001) is itself an important finding with planning implications, as lesions requiring fissure crossing are systematically deeper, compounding both risk factors simultaneously. Recent evidence has further highlighted the importance of the anatomical relationship between the target lesion and pleural surface. Deng et al. [23] demonstrated that nodule–pleura interactions and puncture trajectory selection significantly influence PNX occurrence, reinforcing the concept that biopsy-related risk is strongly dependent on pleural disruption and the amount of traversed aerated lung.

Patient age as an independent predictor (OR 1.076 per year) is consistent with prior meta-analytic findings [10,12]. Potential mechanisms include age-related decline in pleural elasticity and repair capacity, increased co-prevalence of emphysema and air-trapping, and reduced respiratory muscular reserve, limiting the patient’s ability to maintain breath-hold during needle manipulation. Notably, age retained significance after adjustment for emphysema, suggesting it captures additional unmeasured biological and physiological determinants beyond parenchymal air-trapping alone.

Emphysema did not retain independent significance in multivariate analysis despite a clinically plausible univariate trend (57.0% vs. 44.7%; *p* = 0.099). This likely reflects a combination of limited statistical power, high baseline prevalence of emphysema in this smoking-heavy cohort (49.2%), binary coding without severity quantification, and partial collinearity with age. Yoon et al. [9] and Huo et al.’s meta-analyses [10] both identified emphysema as an independent predictor in larger cohorts—future studies with CT-densitometric emphysema scoring will resolve whether the binary variable used here was simply insufficiently discriminative. This limitation is further discussed in the Limitations section.

#### 4.2.2. Alveolar Hemorrhage: Incidence and Risk Factors

The alveolar hemorrhage rate of 20.4% is consistent with published ranges (5–27%) [5,8,9,12], though heterogeneity in definitions complicates direct comparison. All cases were managed conservatively, indicating that the clinical burden of this complication in our series was low despite its frequency.

Pleura-to-lesion distance as the dominant hemorrhage predictor (OR 1.768 per 10 mm) is mechanistically straightforward: longer intrapulmonary needle paths traverse greater volumes of vascularised parenchyma, crossing more bronchial and pulmonary arterial branches. The finding that maximum lesion size did not survive multivariate adjustment suggests that lesion diameter is a marker for depth in certain anatomical configurations, and that the depth variable captures the biologically active exposure more precisely. This is consistent with Yoon et al. [9], who similarly found that pleura-to-lesion distance outcompeted lesion size in adjusted hemorrhage models.

The independent fourfold increase in hemorrhage risk associated with RUL apical segment location (OR 4.281; *p* < 0.001) is a noteworthy and novel finding. The apical segment of the RUL presents several anatomically distinct features: proximity to intercostal and subclavian vasculature at the lung apex; a high prevalence of emphysematous bullous disease at the apices in smokers, which fragments the parenchyma and exposes vessels with reduced perivascular support; and the geometric constraints of needle positioning beneath the clavicle and first rib, which frequently force longer or more oblique trajectories. This finding warrants prospective validation and, if confirmed, argues for enhanced post-procedural monitoring following apical RUL biopsies, with particular attention to signs of hemorrhagic progression.

The protective effect of carcinomatous lymphangitis against alveolar hemorrhage (OR 0.359; *p* = 0.030) represents a hypothesis-generating finding that warrants cautious interpretation. The same variable also demonstrated complete protection against chest tube drainage in our cohort (0/73 events vs. 14/165 without lymphangitis; Fisher *p* = 0.006). One potential biological explanation is that carcinomatous lymphangitis involves peribronchovascular tumour infiltration accompanied by a desmoplastic fibrous stromal reaction that may physically reduce microvascular permeability and create a more fibrotic, less hemorrhagic needle track environment. An alternative explanation is confounding by indication: lymphangitis is a marker of advanced, subpleural distributed, heavily vascularised disease that may occupy segments where shorter needle paths are anatomically available, reducing traversal length. The current retrospective design does not permit causal inference or mechanistic discrimination between these two explanations. Prospective studies with systematic measurement of pleura-to-lesion distance stratified by lymphangitis status are needed to determine whether this association reflects a true biological protective effect or an anatomical confound.

### 4.3. Pathophysiological Interpretation

The striking divergence between the PNX and hemorrhage risk factor profiles invites a pathophysiological interpretation. PNX risk is mainly governed by the characteristics of the pleural breach—its size, geometry, and the tissue’s capacity for rapid spontaneous closure. A near-perpendicular needle creates a small, circular pleural defect that heals efficiently; an oblique needle generates an elongated, slit-like tract that remains patent under respiratory pressure cycles. Fissure traversal compounds this by adding a second pleural surface to the injury. Similarly, the effect of patient positioning and biopsy approach appears to be mediated primarily through procedural geometry. Lateral approaches may restrict trajectory optimization due to chest wall curvature and intercostal access limitations, increasing the probability of an oblique pleural entry angle. Therefore, the increased risk observed with certain approaches likely reflects anatomical accessibility and needle trajectory rather than patient position itself. These are trajectory-determined events for which operator decisions are the primary modifiable levers.

Hemorrhage risk, in contrast, is primarily determined by lesion depth within the vascularised parenchyma. Greater traversal distance exposes more bronchopulmonary arterial and venous branches to potential injury, while anatomical factors modulate the density and fragility of the vasculature encountered. Segmental location further modifies this risk by influencing both the anatomical structures encountered and the technical constraints of needle placement. In particular, RUL apical lesions may require trajectories limited by the clavicle, first rib, and narrow apical access window, reducing operator flexibility in selecting the shortest and safest needle path. These anatomical restrictions may increase the likelihood of traversing vulnerable parenchyma or vascular structures, providing a potential explanation for the increased hemorrhage risk observed in this subgroup. Critically, needle angle does not independently predict hemorrhage, because even perpendicular needles must traverse whatever parenchymal distance separates the pleura from the lesion. The two complications are driven by only partially overlapping mechanisms, which explains why attempts to reduce PNX risk by optimizing needle angle do not simultaneously increase alveolar hemorrhage rates.

This mechanistic framework has a practical corollary: the two complications can be risk-stratified and addressed with partially distinct procedural strategies. For PNX prevention, the primary levers are needle trajectory optimization and fissure avoidance. For hemorrhage mitigation, the primary targets are minimizing traversal depth (selecting the shortest available needle path to the lesion margin) and exercising heightened vigilance in anatomically high-risk segments, particularly the RUL apex.

### 4.4. Clinical Implications

The identified determinants can be broadly divided into modifiable and non-modifiable factors. Needle–pleural angle, biopsy approach, and fissure avoidance are operator-dependent and represent the primary targets for active complication reduction. Meanwhile, patient age, lesion depth, anatomical location, and parenchymal background, although not amenable to modification, inform pre-procedural risk stratification and guide post-procedural monitoring decisions.

1. Pre-procedural risk stratification and counselling.

The logistic regression models presented here enable quantitative pre-procedural risk estimation for both primary complications. Operators can identify high-risk profiles prior to the procedure: patients with deep lesions (>22 mm pleura-to-lesion distance), anatomically constrained approach angles (<75°), necessity for fissure traversal, and older age constitute a PNX-prone subgroup warranting explicit informed consent regarding chest tube probability (~18% in this cohort) and pre-notification of the medical or surgical team. ROC curve analysis independently identified a Youden-optimal threshold of 75° for needle–pleural angle, providing data-derived validation for this value as a practical procedural target. As illustrated in Supplementary Figure S2 (Panel A), the predicted probability of PNX at a peripheral lesion (0 mm depth) decreases from approximately 95% at 50° to below 2% at 90°, with the steepest risk reduction occurring between 65° and 80°. Even at intermediate depth (20 mm), optimizing the angle from 65° to 80° reduces predicted PNX probability from approximately 80.4% to 22%, underscoring the magnitude of risk reduction achievable through trajectory planning alone.

For alveolar hemorrhage, pre-procedural risk identification depends on two non-modifiable factors: lesion depth and anatomical location. The Youden-optimal threshold of 22 mm for pleura-to-lesion distance confirmed the clinical relevance of this parameter as a risk stratifier. Patients with RUL apical lesions and anticipated traversal distances exceeding 22 mm represent the highest-risk subgroup for hemorrhagic complications and should be identified before the procedure for increased post-procedural monitoring. As shown in Supplementary Figure S2 (Panel B), the predicted probability of alveolar hemorrhage at 22 mm depth reaches approximately 54% for RUL apical lesions compared to approximately 22% for non-RUL locations, a more than twofold difference attributable solely to segment location independent of depth.

2. Needle angle as a primary modifiable target.

The consistent dose–response association between needle angle and all PNX-related outcomes establishes angle optimization as the single most impactful modifiable intervention available to the operator. Achieving an angle above 75° may not always be anatomically feasible. Notable constraints include RUL apical lesions bounded by the clavicle and first rib, paravertebral lesions limited by the vertebral column, anterior lesions where mediastinal structures or internal mammary vessels may restrict the available trajectory, and posterior upper lobe lesions where scapular positioning limits the intercostal window. In these scenarios, the 75° threshold should be considered a practical reference point for trajectory optimization rather than an absolute requirement, and must be balanced with other procedural considerations such as minimizing pleura-to-lesion distance and avoiding fissures or vascular structures. The analysis demonstrates that even small incremental improvements towards perpendicularity yield measurable risk reductions, as evidenced by the continuous relationship shown in Supplementary Figure S2 (Panel A). This evidence supports the implementation of multi-planar CT reconstruction-based trajectory planning as a routine pre-procedural step, with an explicit angle target documented in the procedure protocol. Operators could use the 75° threshold as an audit criterion: tracking the number of procedures performed above this threshold over time offers a concrete, measurable quality metric directly linked to patient outcomes.

3. Post-procedural monitoring stratification.

The 10 min rim reassessment strategy identified 15 out of 77 PNX patients (19.5%) as progressors—a clinically important group whose PNX required active management. The rim progression model (AUC 0.789) identifies oblique angle and fissure traversal as predictors: these patients derive the greatest benefit from systematic 10 min re-scanning and should be considered for extended observation, even when the initial rim appears modest. Conversely, the 80.5% of PNX patients with stable rims at 10 min represent candidates for ambulatory management of small pneumothoraces in appropriately selected contexts, a strategy that could reduce resource utilization without compromising safety. This risk-stratification approach operationalises the 10 min re-scan not merely as a safety check but as a dynamic triage tool to allocate monitoring intensity.

4. Diagnostic yield and procedural technique.

A predominantly single-pass, non-coaxial technique achieves a diagnostic yield of 84.2% overall (89.1% specimen-adjusted), consistent with the 85–95% range reported across institutional series [6] and closely approximating the 88.9% yield reported by Balasubramanian et al. [5]. The technique reduced the risk of cumulative pleural trauma from multiple passes and did not compromise diagnostic quality compared with coaxial multi-pass alternatives [9,17]. The 1.7% procedure abortion rate is low compared to the 8.6% discontinuation rate reported by Johansen et al. [22]. These findings suggest that procedural standardization, needle selection, and operator experience independently contribute to diagnostic yield, beyond lesion-related factors [5,6,22].

### 4.5. Limitations

This study has several limitations that should be considered when interpreting the findings.

**Retrospective single-centre design.** The retrospective nature of the study introduces inherent risks of selection bias, incomplete data ascertainment, and unmeasured confounding. Consecutive enrolment mitigates referral bias within the study period. However, our cohort reflects a specific demographic characterized by high smoking prevalence and predominantly advanced-stage disease at presentation, which may limit generalisability to populations with different epidemiological profiles. Additionally, the exclusion of cases with incomplete procedural data may have selectively removed patients with aborted or technically complex procedures, potentially underestimating true complication rates. The presented risk models should be considered hypothesis-generating and require external prospective validation before deployment in routine clinical decision-making.

**Events-per-variable constraints and model validation.** Bootstrap internal validation of the PNX and alveolar hemorrhage logistic regression models confirmed minimal optimism (optimism-corrected AUC values of 0.918 and 0.809, respectively), supporting the robustness of these estimates. By contrast, the chest tube drainage and PNX rim progression analyses (EPV 7.0 and 7.5, respectively) were constructed with fewer events per predictor variable than the conventionally recommended threshold of 10. These models are explicitly flagged as exploratory throughout. Although both achieved strong discrimination and calibration, small-sample logistic models carry elevated risks of overfitting and biassed coefficient estimation, and the wide confidence intervals reflect this uncertainty. Statistical conclusions derived from these subgroups must not be used for clinical decision-making without prospective validation in adequately powered cohorts. Prospective series specifically designed to evaluate these rarer endpoints at adequate EPV are required to confirm or refute these findings.

**Binary coding of emphysema without severity grading.** Emphysema was recorded as a binary present/absent variable, without quantitative assessment of extent or severity. This binary system may have contributed to the loss of multivariate significance, despite the well-established association between emphysema severity and PNX risk in larger series [9,10]. Future studies should incorporate a validated quantitative emphysema score to enable dose–response modelling of this clinically important co-variable.

**Absence of operator-level experience stratification.** Operator experience has been identified as a significant determinant of both PNX rate and diagnostic yield in the prior literature [9], and its omission represents a potentially important unmeasured confounder. Less experienced operators may systematically achieve lower needle angles and generate higher complication rates independently of lesion characteristics. Prospective registries should capture a procedure-level operator experience metric to enable adjustment.

**Absence of patient-reported outcomes.** Complications were graded using the SIR adverse event classification [14] based on clinical and imaging criteria. Patient-reported outcomes were not collected. These outcomes are increasingly recognized as clinically meaningful quality metrics in interventional radiology and should be incorporated as pre-specified endpoints in future prospective registries.

**Follow-up duration and delayed complications.** After biopsy, all patients underwent routine post-procedural monitoring, including clinical observation for early detection of complications. Patients remained hospitalized for at least one night after the procedure and were clinically followed during this period. Additional imaging evaluation was performed when clinically indicated, particularly in cases of new or worsening symptoms suggestive of delayed complications. However, systematic follow-up after hospital discharge was not included in the study protocol; therefore, delayed events managed outside our institution may not have been captured. Future prospective studies should incorporate structured follow-up at 24 h, 7 days, and 30 days as pre-specified endpoints.

## 5. Conclusions

In this single-centre retrospective series of 240 CT-guided transthoracic lung biopsies, we identified distinct and mechanistically interpretable independent determinants of PNX and alveolar hemorrhage. The needle–pleural angle was the dominant modifiable predictor of PNX and PNX-related outcomes, with each additional degree towards perpendicularity conferring a consistent, measurable risk reduction. Pleura-to-lesion distance was the primary anatomical determinant shared by both complications. Fissure traversal and patient age independently amplified PNX risk, while RUL apical segment location and absence of carcinomatous lymphangitis independently increased alveolar hemorrhage risk.

The absence of an independent association between needle–pleural angle and hemorrhage risk enables trajectory optimization as a pneumothorax reduction strategy without a hemorrhagic trade-off. These findings support routine angle-based trajectory planning targeting ≥75°, active fissure avoidance during needle path selection, and heightened post-procedural monitoring for patients with deep lesions (>22 mm) or RUL apical location. Prospective multi-centre validation and formal evaluation of angle optimization protocols as complication reduction interventions represent the priority next steps.

## Data Availability

The information is contained within this article in its entirety. For additional information, please feel free to inquire with either the original author or the corresponding author. Public access to data is restricted as a result of patient privacy standards that regulate the handling of clinical data.

## Supplementary Materials

The following supporting information can be downloaded at: https://www.mdpi.com/article/10.3390/diagnostics16121848/s1, Figure S1: Decision curve analysis evaluating the clinical utility of the developed predictive models. Panel A illustrates the net benefit of the pneumothorax prediction model across different threshold probabilities compared with the “treat all” and “treat none” strategies. Panel B shows the corresponding decision curve analysis for the alveolar hemorrhage prediction model, demonstrating the potential clinical value of the model across clinically relevant thresholds; Figure S2: Predicted probability curves for the main continuous predictors identified in the multivariate models. Panel A illustrates the relationship between the needle–pleural angle and the predicted probability of pneumothorax according to lesion depth, highlighting the protective effect of a near-perpendicular trajectory (≥75°). Panel B shows the association between pleura-to-lesion distance and the predicted probability of alveolar hemorrhage, stratified by lesion location (apical versus non-apical).
