# Supplementary figures and images for: Determinants of Pneumothorax and Alveolar Hemorrhage After CT-Guided Lung Biopsy
Source: Diagnostics (Basel). 2026 Jun 15;16(12):1848. doi: 10.3390/diagnostics16121848 (PMC13298841; doi:10.3390/diagnostics16121848)

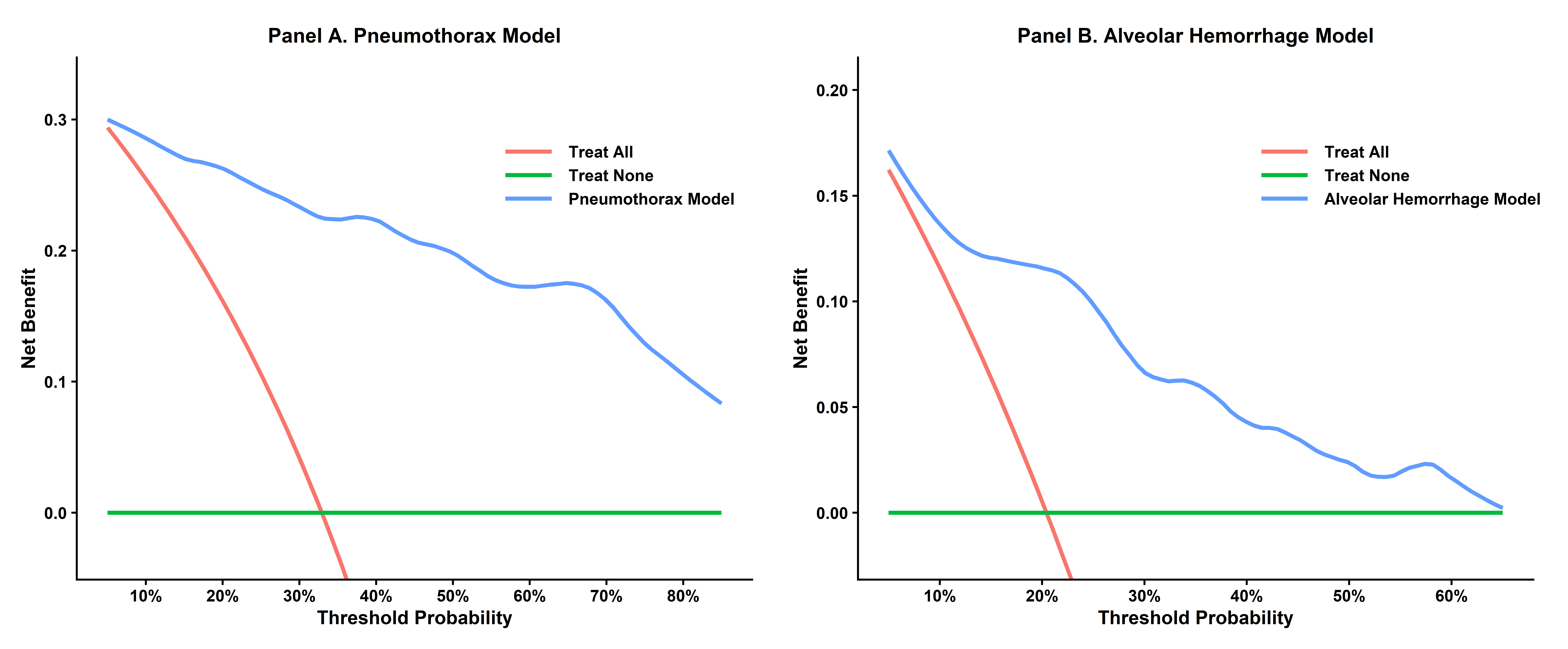

Supplement: Supplementary file 1 [file diagnostics-16-01848-s001.zip › Supplementary Figure S1.tif]

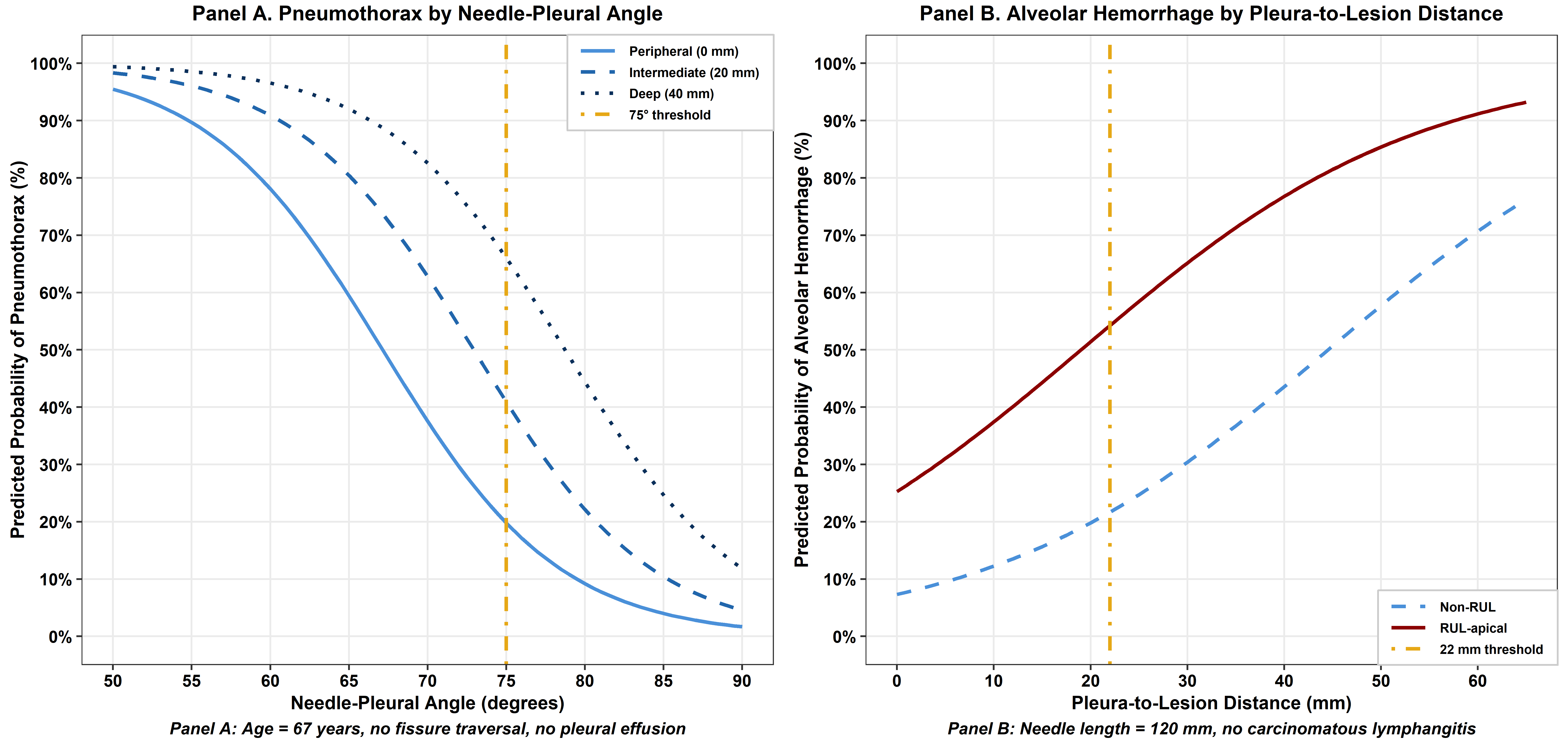

Supplement: Supplementary file 1 [file diagnostics-16-01848-s001.zip › Supplementary Figure S2.tif]
